# Pneumocephalus and Pneumorrhachis Following Titanium Rib Implant: A Case Report and Literature Review

**DOI:** 10.2174/0115734056375842250109093802

**Published:** 2025-01-13

**Authors:** Yusuf Koksal, Sefer Burak Aydin

**Affiliations:** 1 Consultant in Emergency Medicine, WIC Clinic, Primary Health Care Corporation, Doha, Qatar

**Keywords:** Pneumocephalus, Pneumorrhachis, Rib implant, Thoracic surgery, Postoperative complication, Computed tomography, Case report, Literature review

## Abstract

**Introduction:**

Pneumocephalus and pneumorrhachis are rare postoperative complications, commonly occurring within a few days to months after spinal surgery. They are very rarely reported after thoracic surgeries. This case highlights a unique presentation in the emergency department involving headache and vomiting caused by late complications following thoracic surgery with a titanium rib implant.

**Case Presentation:**

A 64-year-old male presented to the emergency department with headache and vomiting without fever since prior 1 week. He had a history of left lower lobectomy and thoracic wall reconstruction with a titanium rib implant 40 days earlier due to epidermoid lung cancer. Computed tomography imaging of head and thorax revealed bilateral pneumocephalus and extensive pneumorrhachis. After removal of the rib implant and dural repair, the patient fully recovered.

**Conclusion:**

This case underscores the importance of early imaging and diagnosis in patients presenting with neurological symptoms following thoracic surgery and emphasizes the need for enhanced monitoring protocols for patients with titanium implants.

## 1 INTRODUCTION

Pneumocephalus and pneumorrhachis are rare but significant postoperative complications that can arise following various surgical procedures, particularly those involving the cranial and spinal regions, and commonly occur within a few days to months after surgery [1]. Pneumocephalus refers to the presence of air within the cranial cavity, while pneumorrhachis denotes air within the spinal epidural or subarachnoid space and is typically associated with trauma or neurosurgery [2, 3]. Their occurrence after thoracic surgeries is infrequently reported [4, 5]. According to a review, spontaneous pneumorrhachis has been reported in 49 cases, with only six cases associated with concurrent pneumocephalus in the literature [6]. Common triggers include upper respiratory infections, asthma exacerbations, emphysematous pyelonephritis, vomiting due to diabetic ketoacidosis, and cocaine use, with rare causes, such as the Valsalva, self-induced vomiting, and cerebrospinal fluid leaks [6-8].

The rarity of these complications following thoracic surgeries, particularly with titanium rib implants, underscores the need for clinicians to remain vigilant, as late-onset neurological symptoms can develop even weeks after surgery, such as in our case that occurred after 40 days postoperatively.

This case report and literature review presents a unique instance of headache and vomiting admitted to the emergency department resulting from pneumorrhachis due to a titanium rib implant complication during thoracic wall reconstruction, and provides an overview of 16 previously published cases in the English literature that have documented similar complications following thoracic procedures.

## 2 CASE PRESENTATION

A 64-year-old male presented to the Emergency Department (ED) with a one-week history of persistent headache and vomiting without fever. His medical history included Chronic Obstructive Pulmonary Disease (COPD), epidermoid lung cancer (stage 2b), and thoracic surgery (left lower lobectomy and thoracic wall reconstruction with a titanium rib implant) performed 40 days earlier. He was in good condition following the surgery, experiencing only occasional episodes of emesis. One week prior to admission, vomiting recurred and was followed by the onset of a mild headache that progressively worsened each day. On the day of his admission to the Emergency Department (ED), he described experiencing the most severe headache and vomiting of his life. He had no history of trauma. He was a heavy smoker who quit smoking 10 years ago. His family history revealed an uncle who died from lung cancer, with no known genetic disorders.

The physical examination parameters were as follows: blood pressure: 123/68 mmHg, heart rate: 68/minute, fever: 36°C, and O_2_ saturation: 94%. During a neurological examination, the Glasgow coma scale score was found to be 15, and the patient was alert, fully oriented, and had no signs of focal neurological deficits, being in good general condition. He had no nuchal rigidity and only had horizontal nystagmus mimicking a central cause.

**Fig. (1a-h):**
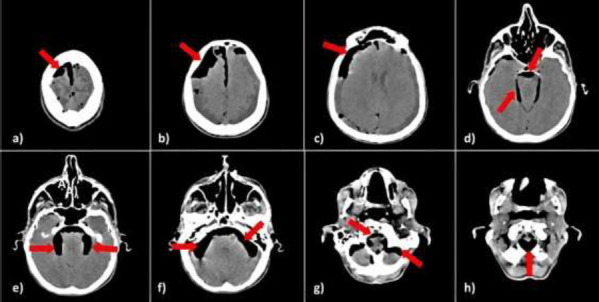
Bilateral pneumocephalus and air density in the foramen magnum observed in cranial CT scans. A subdural effusion could be seen in all sections. This finding supports the diagnosis of post-surgical pneumocephalus.

**Fig. (2):**
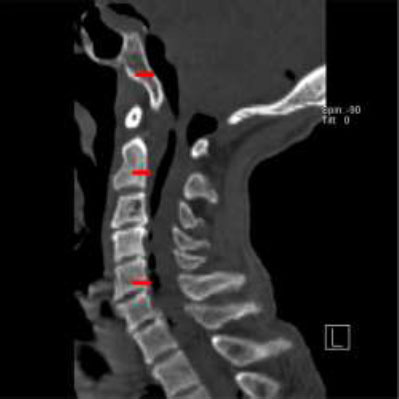
Air densities visible in the cervical CT scan extending from the clivus to thoracic vertebrae (pneumorrhachis), indicative of subarachnoid pleural fistula.

**Fig. (3a,b):**
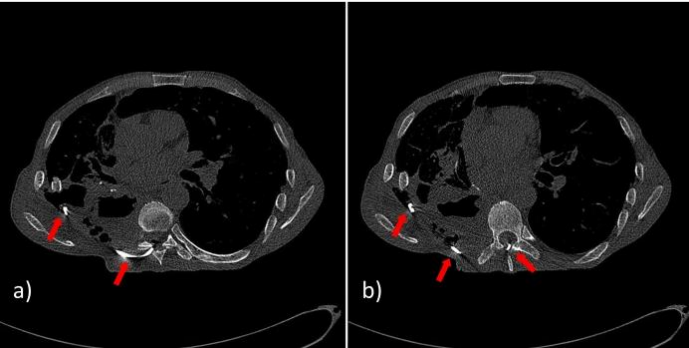
Axial view of thoracic CT showing titanium costa material protruding into the vertebral epidural space. In these sections, air in the dural area could be seen too.

**Fig. (4a,b):**
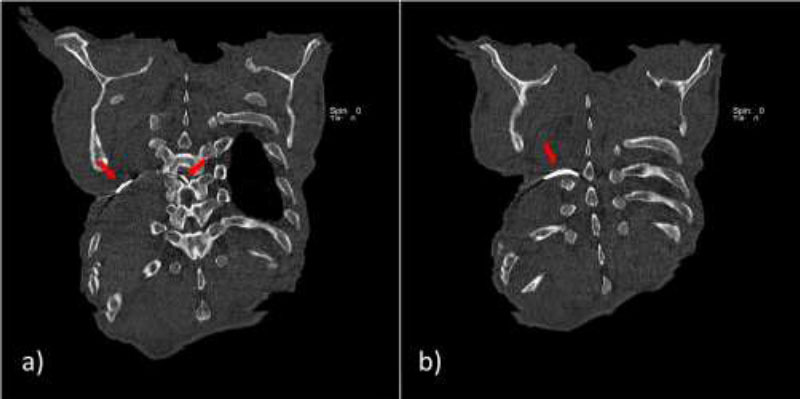
Coronal view of thoracic CT illustrating the titanium costa material in the vertebral epidural space.

**Fig. (5a,b):**
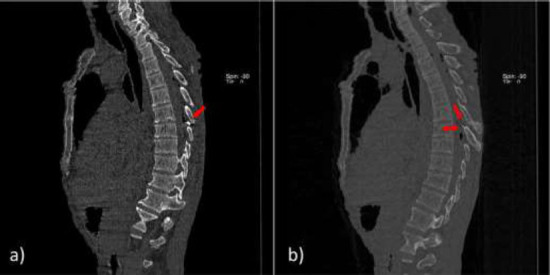
Sagittal view of thoracic CT depicting the titanium implant’s trajectory in the spinal epidural region.

Laboratory results showed leukocytosis (13,500/mm^3^) and elevated CRP levels (3.352 mg/dL). Cranial Computed Tomography (CT) was performed in the ED to exclude intracranial hemorrhage and masses, revealing bilateral pneumocephalus (Fig. **1**). To identify the cause, further cervical and thoracic CT scans were required; however, convincing the patient for additional imaging and coordinating with the radiology department posed a challenge. This resulted in a delay, and the subsequent scans were conducted three hours after the initial CT. Cervical imaging revealed pneumorrhachis extending from the clivus to the thoracic vertebrae (Fig. **2**), while thoracic imaging identified a titanium implant breaching the epidural space (Figs. **3**-**5**), pinpointing the root of the air leak into the arachnoid space.

**Fig. (6a,b):**
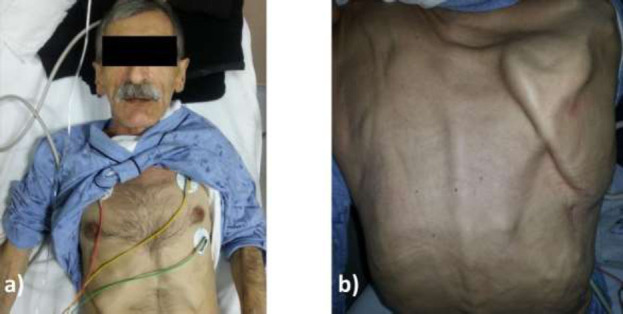
Anterior and posterior clinical photographs of the patient, demonstrating the pre-operative physical state and inspection of the thoracic area.

Management included Intravenous (IV) ceftriaxone 2 grams daily, pain and vomiting control with paracetamol 1000 mg and ondansetron 8 mg IV as needed, head elevation to 30 degrees, and early surgical removal of the titanium rib implant with dural repair. Preoperative physical views of the patient’s anterior and posterior thoracic area were documented (Fig. **6**). The patient made a full recovery and was discharged on postoperative day 15 without any adverse events.

Follow-up imaging was not performed, leaving the resolution of arachnoid air uncertain. While the patient’s symptoms resolved clinically, adherence to and tolerability of the interventions were not formally assessed. However, no adverse effects or complications were reported during hospitalization, and clinical recovery was successfully achieved.

### 2.1 Patient Perspective

The patient reported the following statement: “I never expected such severe complications after surgery. I was feeling good till last week. The persistent headache and vomiting were unbearable, but the emergency team quickly identified the issue and explained the severity. Though the idea of another surgery was daunting, their prompt action and care reassured me. Now I’m fully recovered and grateful for their expertise.”

## 3 DISCUSSION

Postoperative pneumocephalus and pneumorrhachis are rare but serious complications following surgery. In our case, the titanium rib implant likely caused a dural tear and pleura-arachnoid fistula, facilitating sterile air migration into the craniospinal axis, a mechanism that, as reported in the literature, can also arise from other causes, like trauma and spinal surgery, and may manifest as a late complication [11, 25]. Some reports suggest that symptom onset can occur weeks to months postoperatively, aligning with the timeline observed in our patient [26]. A recent study by Bae *et al*. proposed that pneumorrhachis may stem from intraoperative dural injury. Cerebrospinal fluid leakage through the dural defect can induce cerebral hypotension. A negative pressure gradient occurs, which prompts retrograde air migration into the spinal canal. In advanced cases, as we observed in our case, this phenomenon may progress to pleural-arachnoid fistula formation and pneumocephalus due to a substantial influx of air. Persistent pneumocephalus can evolve into tension pneumocephalus, increasing the likelihood of intracranial hemorrhage [27]. Similar instances of pneumocephalus and pneumorrhachis have been documented in the literature following thoracic procedures (Table **1**). The 16 case reports we were able to find on English online databases have reported similar complications after thoracic procedures; a total of 7 cases presented with pneumocephalus alone, 4 patients had isolated pneumorrhachis, and 5 cases manifested both pneumocephalus and pneumorrhachis. The patients’ ages ranged widely (23 to 89 years). A total of 7 female and 9 male patients reported similar complications in the literature. 11 patients underwent surgical repairs, while 5 patients received conservative treatment from these 16 chronological reports. Two patients had fatal outcomes despite these interventions and 2 patients’ outcomes were not reported by the authors [9-24]. These findings suggest that pneumocephalus and pneumorrhachis, although rare, remain critical postoperative complications following thoracic procedures. However, multiple etiologies have been described in thoracic surgeries. To our knowledge, no prior case has involved a titanium rib implant as the source of a dural injury and sterile extended duration of air leak, caused by pneumocephalus and pneumorrhachis. This etiology diverges from the predominantly reported causes, which are often traumatic, infectious, or idiopathic in nature. The unusual sterile air migration resulting from the implant breach underscores the necessity for clinicians to consider atypical sources of air leaks when investigating postoperative neurological complications. The fatal outcomes reported in the literature highlight the risk of severe neurological morbidity if dural breaches are not promptly detected and managed (Table **1**). The application of high-resolution imaging, as demonstrated in our case, was instrumental in identifying the air migration path. Advances in medical image processing, such as feature-preserving mesh networks and spatial-asymmetric attention models, offer potential enhancements for detecting subtle postoperative complications, like the cases we observed with delayed diagnosis in the literature [13]. For instance, the feature-preserving mesh network framework introduced by Imran *et al*. offered advanced segmentation of retinal vascular structures, illustrating how deep learning can effectively map complex anatomical regions, a principle that could be adapted for pneumocephalus and pneumorrhachis root cause detection [28]. Additionally, deblurring algorithms, such as the MedDeblur framework proposed by Sharif *et al*., utilize residual dense spatial-asymmetric attention to enhance visualization in challenging scenarios where imaging artifacts or subtle findings may obscure key structures [29]. By leveraging spatial-asymmetric attention to concentrate computational resources on key anatomical regions, these advanced methods can detect disruptions or implant-related complications more precisely. Therefore, they may enhance diagnostic accuracy in postoperative imaging. They also offer the potential for refining the detection of small air pockets in cranial and spinal spaces. However, further studies are warranted to optimize these techniques for postsurgical monitoring. Our case report and literature review can help broaden the understanding of potential etiologies and mechanisms for such complications and emphasize the critical role of high-resolution imaging and interdisciplinary approaches in identifying these cases. Our report is, however, limited by a lack of long-term follow-up and imaging, which prevented determining when the air in the arachnoid space resolved. Clinicians must maintain a high index of suspicion for dural injuries in patients presenting with unexplained late neurological symptoms after thoracic procedures, as according to literature, early diagnosis and surgical intervention can significantly improve outcomes [30, 31]. Prompt imaging, a high index of clinical suspicion, and interdisciplinary collaboration are paramount to ensure timely diagnosis and prevent bad outcomes, particularly in patients with novel thoracic implants who present even with late-onset neurological symptoms.

## CONCLUSION

Emergency physicians, surgeons, and radiologists should maintain a heightened level of clinical vigilance for potential dural injuries in patients who develop unexpected or delayed neurological symptoms following thoracic surgery. Enhanced postoperative monitoring protocols, particularly for individuals with titanium implants, combined with high-resolution imaging, are essential for timely recognition.

## Figures and Tables

**Table 1:** Reported cases of pneumocephalus and/or pneumorrhachis after thoracic surgeries/procedures.

**Study/Refs.**	**Year**	**A/G**	**PC**	**PR**	**Cause**	**Treatment** **(outcome)**
Malca *et al*. [9]	1995	75/M	+	-	Pneumothorax following surgical resection of malignancy *via* thoracotomy leading to dural injury	Surgical intervention (deceased)
Singh *et al*. [10]	1999	40/M	+	-	Thoracic tumor resection resulting in dural injury	Surgical intervention (survived)
Lin *et al*. [11]	2000	59/F	+	+	Pleuro-subarachnoid fistula secondary to posterior isola rod fixation and T3 bone grafting	Conservative management (survived)
Ristagno *et al*. [12]	2002	60/M	-	+	Bronchopleural-subcutaneous fistula following lobectomy for lung carcinoma	Surgical intervention (survived)
Ozturk *et al*. [13]	2009	23/F	+	-	Misplacement of a pedicle screw during thoracolumbar scoliosis surgery resulting in dural injury	Surgical intervention (survived)
Son *et al*. [14]	2011	45/F	+	+	Broncho-paraspinal fistula caused by metallic instrumentation during thoracic spinal surgery	Surgical intervention (unknown)
Müller *et al*. [15]	2015	69/M	+	-	Pleura-arachnoid fistula due to dural injury during posterior thoracic wall resection	Surgical intervention (survived)
Sugimoto *et al*. [16]	2015	79/M	+	-	Pleural-subarachnoid fistula following T2–T3 rib resection and partial vertebrectomy for lung cancer	Surgical intervention (survived)
Mattei *et al*. [17]	2015	49/F	-	+	Dural injury caused by percutaneous vertebroplasty at T8	Surgical intervention (survived)
Lee *et al*. [18]	2016	47/F	+	+	Dural injury caused during posterior decompression (T11–L2) and thoracic spine surgery	Conservative management (survived)
Yazgan *et al*. [19]	2020	70/M	+	-	Dural defect caused by Pancoast tumor resection	Conservative management (survived)
MacDonald *et al*. [20]	2022	27/M	-	+	Air leak caused by thoracic epidural catheter placement during laparotomy for Crohn's disease	Conservative management (survived)
Sonfack *et al*. [21]	2023	69/M	+	+	Dural injury caused by thoracoscopic pleurodesis for recurrent pneumothorax	Surgical intervention (survived)
Brueske *et al*. [22]	2024	69/F	+	-	Air leak caused by epidural catheter (T5–T6) placement and right-sided pneumonectomy	Surgical intervention (deceased)
Kang *et al*. [23]	2024	89/M	-	+	Thoracic epidural block caused dural injury and air leak	Surgical intervention (unknown)
Lipinski *et al*. [24]	2024	53/F	+	+	Bronchopleural fistula resulting in pneumocephalus and pneumorrhachis	Surgical intervention (survived)
**Presented case**	**2024**	**64/M**	**+**	**+**	**Pleura-arachnoid fistula caused by titanium rib implant**	**Surgical removal of the implant and dural repair (survived)**

**Note: **A: Age, G: Gender, PC: Pneumocephalus, PR: Pneumorrhachis, T: Thoracic, L: Lumber.

The primary diagnoses were pneumocephalus and/or pneumorrhachis, predominantly arising from thoracic surgical or procedural complications. Treatment ranged from conservative management to surgical intervention, and some cases ended in mortality.

## Data Availability

The data supporting the findings of this article will be available from the corresponding author [Y.K] upon reasonable request.

## LIST OF ABBREVIATIONS

COPD: Chronic obstructive pulmonary disease
ED: Emergency department
IV: Intravenous
CT: Computed tomography

## STANDARDS OF REPORTING

CARE guidelines were followed.

## References

[1] Turgut M., Akyüz O. (2007). Symptomatic tension pneumocephalus: An unusual post-operative complication of posterior spinal surgery.. J. Clin. Neurosci..

[2] Katz D.S., Groskin S.A., Wasenko J.J. (1994). Pneumorachis and pneumocephalus caused by pneumothorax and multiple thoracic vertebral fractures.. Clin. Imaging.

[3] Jelsma F., Moore D.F. (1954). Cranial aerocele.. Am. J. Surg..

[4] Machetanz K., Leuze F., Mounts K., Trakolis L., Gugel I., Grimm F., Tatagiba M., Naros G. (2020). Occurrence and management of postoperative pneumocephalus using the semi-sitting position in vestibular schwannoma surgery.. Acta Neurochir..

[5] Sheikh F., Nasir J., Khalid A., Saleem R., Sheikh F., Rauf A. (2021). Single burrhole craniostomy with subdural placement of foley catheter for drainage of chronic subdural hematoma.. Pak. J. Neurol. Surg..

[6] Alampoondi Venkataramanan S.V., George L., Sahu K.K. (2021). Spontaneous pneumorachis – A case-based review.. J. Asthma Allergy.

[7] Gâta A., Toader C., Trombitaș V.E., Ilyes A., Albu S. (2020). Endoscopic skull base repair strategy for CSF leaks associated with pneumocephalus.. J. Clin. Med..

[8] Gönül E., Izci Y., Sali A., Baysefer A., Timurkaynak E. (2000). Subdural and intraventricular traumatic tension pneumocephalus: Case report.. Minim. Invasive Neurosurg..

[9] Malca S.A., Roche P.H., Touta A., Pellet W. (1995). Pneumocephalus after thoracotomy.. Surg. Neurol..

[10] Singh R.S., Pathak A. (1999). Tension pneumocephalus after excision of posterior mediastinal mass.. Ann. Thorac. Surg..

[11] Lin M.B., Cheah F.K., Ng S.E., Yeo T.T. (2000). Tension pneumocephalus and pneumorachis secondary to subarachnoid pleural fistula.. Br. J. Radiol..

[12] Ristagno R.L., Hiratzka L.F., Rost R.C. (2002). An unusual case of pneumorrhachis following resection of lung carcinoma.. Chest.

[13] Ozturk E., Kantarci M., Karaman K., Cinar Basekim C., Kizilkaya E. (2006). Diffuse pneumocephalus associated with infratentorial and supratentorial hemorrhages as a complication of spinal surgery.. Acta Radiol..

[14] Son S., Kang D.H., Choi D.S., Choi N.C. (2011). A case of broncho-paraspinal fistula induced by metallic devices: Delayed complication of thoracic spinal surgery.. J. Korean Neurosurg. Soc..

[15] Müller I, Tönnies M, Pfannschmidt J, Kaiser D. (2015). Pneumocephalus following thoracic surgery with posterior chest wall resection.. Thorac. Cardiovasc. Surg. Rep..

[16] Sugimoto S., Tanaka M., Suzawa K., Nishikawa H., Toyooka S., Oto T., Ozaki T., Miyoshi S. (2015). Pneumocephalus and chylothorax complicating vertebrectomy for lung cancer.. Ann. Thorac. Surg..

[17] Mattei T.A., Rehman A.A., Dinh D.H. (2015). Acute spinal subdural hematoma after vertebroplasty: A case report emphasizing the possible etiologic role of venous congestion.. Global Spine J..

[18] Lee G.S., Lee M.K., Kim W.J., Kim H.S., Kim J.H., Kim Y.S. (2016). Pneumocephalus and pneumorrhachis due to a subarachnoid pleural fistula that developed after thoracic spine surgery.. Korean J. Spine.

[19] Yazgan S., Gürsoy S., Üçvet A., Acar A., Samancılar Ö., Özer F. (2020). What you donâ€™t want to see after a superior sulcus tumor resection?: A tension pneumocephalus.. Current Thoracic Surgery.

[20] MacDonald S., Mukhida K. (2022). Pneumorrhachis complicating acute pain management using a thoracic epidural catheter.. Dalhousie Med. J..

[21] Sonfack D.J.N., Tarabay B., Shen J., Wang Z., Boubez G., Shédid D., Yuh S.J. (2023). Pneumorrhachis and pneumocephalus resulting from pneumothorax: Illustrative case.. J. Neurosurg. Case Lessons..

[22] Brueske B., He C., Chauhan V. (2024). Devastating neurological outcome following a subarachnoid-pleural fistula during thoracic surgery.. Cureus.

[23] Kang K.B., Kim Y.B., Shin Y.B., Cho S.S., Lee E.D. (2024). Progressive paraplegia after upper thoracic epidural block-related pneumorachis: A case report.. Int. J. Surg. Case Rep..

[24] Lipinski A.W., Smith M.V., Wannamaker E.J., Timpone V.M. (2024). Symptomatic Pneumorrhachis from bronchial-subarachnoid fistula.. Diagnostics.

[25] Dabdoub C., Salas G., Silveira E., Dabdoub C. (2015). Review of the management of pneumocephalus.. Surg. Neurol. Int..

[26] Schuchert M.J., Myers T.G., DeGraft-Johnson J., Bejjani G.K., Luketich J.D., Landreneau R.J. (2009). Pneumocephalus after resection of a lung cancer with posterior chest wall involvement.. Ann. Thorac. Surg..

[27] Bae Y., Yon D.K., Lee S.W. (2024). Cerebrovascular complications in spinal fusion surgery: A nationwide 8-year follow-up study in South Korea.. Clin. Nurs. Res..

[28] Imran S.M.A., Saleem M.W., Hameed M.T., Hussain A., Naqvi R.A., Lee S.W. (2023). Feature preserving mesh network for semantic segmentation of retinal vasculature to support ophthalmic disease analysis.. Front. Med..

[29] Sharif S.M.A., Naqvi R.A., Mehmood Z., Hussain J., Ali A., Lee S.W. (2022). MedDeblur: Medical image deblurring with residual dense spatial-asymmetric attention.. Mathematics.

[30] Gader G., Rkhami M., Daghfous A., Zouaghi M., Zammel I., Badri M. (2022). Pneumocephalus after posterior fossa surgery in prone position: Is that any clinical effect?. Int. J. Surg. Case Rep..

[31] Kopelovich J.C., de la Garza G.O., Greenlee J.D.W., Graham S.M., Udeh C.I., O’Brien E.K. (2012). Pneumocephalus with BiPAP use after transsphenoidal surgery.. J. Clin. Anesth..

